# PD199 Global Collaboration Post-Brexit: Do Project Orbis And The Access Consortium Improve Access In The UK Compared With Europe?

**DOI:** 10.1017/S0266462324004215

**Published:** 2025-01-07

**Authors:** Aigun Gassanova, Katherine Leong, Richard Macaulay

## Abstract

**Introduction:**

As the newly independent regulator for medicines in the UK post-Brexit, the Medicines and Healthcare products Regulatory Agency (MHRA) has entered international regulatory collaborations, notably Project Orbis (PO) and the Access Consortium (AC), to help expedite marketing authorizations. This research compared regulatory and reimbursement outcomes for medicines approved in Great Britain through PO and the AC with corresponding outcomes from the EU4Health Programme.

**Methods:**

Products approved by the MHRA through PO and the AC were identified from the PO and New Active Substance Work Sharing Initiative websites, respectively. Marketing authorization dates and reimbursement outcomes were obtained from the respective regulatory and health technology assessment body websites on 20 November 2023.

**Results:**

A total of 21 products have been authorized by the MHRA through either PO (17/21) or the AC (4/21); 17 of 21 have been authorized in Europe. Approval by the MHRA was 54 days earlier on average than for the European Medicines Agency. The results for various countries were as follows:

- England: 14 of 21 (67%) products assessed (9/14 [64%] recommended);
- Scotland: 14 of 21 (67%) products assessed (11/14 [79%] recommended);
- France: 14 of 21 (67%) products assessed (2/14 [14%] received Amélioration du Service Médical Rendu level I to III);
- Germany: 14 of 21 (67%) products assessed (5/14 [36%] achieved additional benefit);
- Italy: 10 of 21 (48%) products assessed (9/10 [90%] reimbursed); and
- Spain: 10 of 21 (48%) products assessed (7/10 [70%] reimbursed).

Days from regulatory approval to HTA were 306 (Scotland), 308 (France), 310 (UK), 330 (Germany), 398 (Spain), and 404 (Italy).

**Conclusions:**

Compared with the EU4Health Programme, PO and the AC have enabled earlier marketing authorizations for medicines in the UK by an average of seven weeks. However, the proportion of therapies reimbursed and the delay from marketing authorization to reimbursement were comparable or more favorable for UK health technology assessment bodies. Thus, the UK may become a first-to-launch European market for certain therapies through PO and the AC.

